# Association of polymorphism of *PTPN* 11 encoding SHP-2 with gastric atrophy but not gastric cancer in *Helicobacter pylori* seropositive Chinese population

**DOI:** 10.1186/1471-230X-12-89

**Published:** 2012-07-12

**Authors:** Jing Jiang, Zhi-Fang Jia, Fei Kong, Mei-Shan Jin, Yin-Ping Wang, Suyan Tian, Jian Suo, Xueyuan Cao

**Affiliations:** 1 Division of Clinical Epidemiology, Jilin University First Hospital, Changchun, China; 2 Division of Pathology, Jilin University First Hospital, Changchun, China; 3 Department of Gastric and Colorectal Surgery, Jilin University First Hospital, Changchun, China

## Abstract

**Background:**

The interaction between Src homology 2 domain-containing protein tyrosine phosphatase (SHP-2) of gastric epithelial cells and cagA from *H*. *pylori* plays a crucial role in developments of gastric atrophy and gastric cancer. This study aimed to investigate the association of haplotype tagging SNPs (htSNPs) in the *PTPN11* gene encoding SHP-2 with gastric atrophy and gastric cancer in Chinese population.

**Methods:**

The subjects comprised 414 patients with gastric cancer, 109 individuals with gastric atrophy and 923 healthy controls. Blood was collected from October 2008 to October 2010. Five htSNPs rs2301756, rs12423190, rs12229892, rs7958372 and rs4767860 from the *PTPN11* gene were selected and genotyped by Taqman assay. Serum Ig G antibodies to *H*. *pylori* were detected by ELISA. Gastric atrophy was screened by the levels of serum pepsinogenIandII, and confirmed by endoscopy and histopatholgical examinations. Odds ratio (ORs) and 95% confidence intervals (CIs) were calculated by a multivariate logistic regression.

**Results:**

Among *H*. *pylori* seropositive subjects, age and gender-adjusted OR of gastric atrophy was 2.47 (95%CI 1.13-4.55, *P* = 0.02) for CC genotype compared with CT/TT genotypes, suggesting a recessive model of genetic risk for rs12423190. The prevalence of *H*. *pylori* seropositivity were significantly higher in groups of gastric cancer and gastric atrophy compared to the control group (70.3% vs. 75.2% vs. 49.7%, *P* <0.001). However, the distributions of genotypes and haplotypes in patients with gastric cancer were not significantly different from healthy controls.

**Conclusions:**

Our study provides the first evidence that rs12423190 polymorphism of the *PTPN11* gene is significantly associated with an increased risk of gastric atrophy in *H*. *pylori* infected Chinese Han population, suggesting that rs12423190 polymorphism could be used as a useful marker of genetic susceptibility to gastric atrophy among *H*. *pylori* infected subjects. The biological roles of this polymorphism require a further investigation.

## Background

Gastric cancer is the most common malignancy of gastrointestinal tract in East Asian populations and the third most common cause of cancer-related deaths in China [1,2]. *Helicobacter pylori* (*H*. *pylori*) infection has been established as a major risk factor for gastric cancer, through the induction of gastric atrophy and progression of precancerous lesions by numerous studies [3,4]. Although *H*. *pylori* is estimated to inhabit at least half of the world’s human population, just few subjects develop to gastric precancerous lesions and adenocarcinoma. The extent of gastric damages induced by *H*. *pylori* infection seems to vary from one subject to another, suggesting that the combination of host genetic traits and bacterial virulence plays important roles in long-term outcomes of *H*. *pylori* infection [5-7].

Several studies have provided evidences that infection with cagA-positive *H*. *pylori* associates with higher grades of gastric inflammation and is more virulent than the cagA-negative strains [8]. The CagA protein is delivered into gastric epithelial cells via the bacterial type IV secretion system, where it undergoes tyrosine phosphorylation by Src and Abl kinases. Tyrosine-phosphorylated CagA then acquires capability to interact with and deregulate SHP-2 phosphatase, a bona-fide oncoprotein [9]. The formation of cagA/SHP-2 complex induces abnormal proliferation and migration of gastric epithelial cells, consequently resulting in gastric atrophy and gastric carcinoma [10-12]. In addition, gain-of-function mutations of the SHP-2 have recently been found in human malignancies [13-15]. Kim et al also revealed that gastric cancers displayed higher levels of SHP-2 protein compared to normal cells, suggesting that neo-expression of this signalling protein in cells might play a role in the gastric carcinogenesis [16]. Since the protein-tyrosine phosphatase nonreceptor-type 11 (*PTPN11*) gene encodes protein containing two tandem Src homology-2 domains, which function as phospho-tyrosine binding domains. SHP-2 closely interacts with the CagA protein, therefore, it is speculated that functional polymorphisms in the *PTPN11* may mediate the interaction of this protein with its substrates and affect its regulatory role in various cell signalling events, such as mitogenic activation, metabolic control, transcription regulation, cell migration, and malignant transformation in *H*. *pylori* infected subjects.

The *PTPN11* gene is on chromosome 12, containing 16 exons. Several single–nucleotide polymorphisms (SNPs) rs11066322, rs11066320 and rs2301756 have been identified in Caucasian females to be associated with apoB levels and LDL-C levels [17]. Another study demonstrated that the rs11066322 was associated with increased plasma HDL-C levels [18]. These results suggested that genetic variants influencing SHP-2 activities may modulate biological functions of the protein. In gastric cancer, Japanese group has found that a prevalent SNP in intron3 (rs2301756) was associated with an increased risk of gastric atrophy in Japanese population with *H*. *pylori* infection [19-22]. The aim of the present study is to determine whether polymorphisms of *PTPN11* gene are associated with clinical outcomes of *H*. *pylori* infection in Chinese population.

## Methods

### Study populations

Four hundred and fourteen Gastric cancer cases were selected from the department of gastric and colorectal surgery, the First Hospital, Jilin University, from 2008 to 2010. All patients underwent tumor resection with histologically confirmed diagnosis of gastric adenocarcinoma. The gastric atrophy individuals and health controls were recruited from the healthy check-up centre of the same hospital from 2009 to 2010. A total 1080 persons (630 males and 450 females, aged 35 to 80 years old) participated in the study without history of cancer. The examinees were Han inhabitants in Changchun city. The informed consent was obtained from all subjects and the study protocol was approved by the ethics committee of the first affiliated hospital, Jilin University. The examinees received serum anti-*H*. *pylori* IgG titre and pepsinogen examinations for screening *H*. *pylori* infection and gastric atrophy.

### Tests for *H. pylori* infection and diagnosis of gastric Atrophy

Serum immunoglobulin (Ig) G antibodies to *H*. *pylori* were detected by enzyme-linked immunosorbent assay (ELISA) using *H*. *pylori* -IgG ELISA kit (Biohit, Helsink, Finland). The antibody titres were defined by optical density (OD) values according to the manufacturer’s protocol and titres higher than the cut off value of 30EIU, were considered as positive for *H*. *pylori* infection. PepsinogenIand II (PGIand PGII) in serum were measured using ELISA kits (Biohit, Helsink, Finland). For screening gastric atrophy, we decided to use cut-off points of <82.3 ng/ml for PGIand <6.05 for PGI/PGIIratio due to no universally accepted cut-off points for dichotomising PGI or the PGI/PGII ratio, which have been validated against histological confirmatory studies for gastric atrophy [23]. The kit quality control samples showed Coefficient of variations (CVs) of 4.5, 4.3 and 4.7% for *H*. *pylori*, PGI and PGII, respectively. All suspected cases with gastric atrophy by serum screening were re-determined by endoscopic, biopsy and histological examinations for a definite diagnosis.

### Selection of tagging SNPs

**Figure 1:**
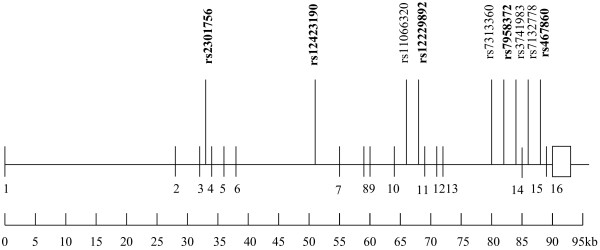
**Genomic map of *PTPN11 gene. *** Validated SNPs are illustrated and htSNPs genotyped in the present study are indicated in bold. Vertical Lines and a box represent exons.

The principal hypothesis underlying this experiment is that one or more common SNPs in *PTPN11* gene are associated with an altered risk of gastric atrophy and/or gastric cancer. Thus, the aim of the SNP tagging is to identify a set of SNPs that efficiently tags all known SNPs. We postulate that such SNPs are also likely to tag any hitherto unidentified SNPs in *PTPN11* gene. Haplotype tagging SNPs (htSNPs) were selected from the Han Chinese data in the HapMap Project (06-02-2009 HapMap) using the SNPbrowser™ Software v4.0 to capture SNPs with a minimum minor allele frequency (MAF) of 0.05 with a pair-wise r square of 0.8 or greater [24]. There are 9 SNPs at minor allele frequencies >0.05 in the *PTPN11* gene in Chinese on HapMap, all of which are located in non-coding regions of the *PTPN11* gene (Figure 1). Finally, five htSNPs rs2301756, rs12423190, rs12229892, rs7958372 and rs4767860 were selected and four of them are shown to be in a complete linkage disequilibrium (LD) with Tag SNP (D’ = 1 and r^2^ >0.8). 

### Genotyping

Genomic DNA from whole blood was extracted with an AxyPrep blood Genomic DNA extraction Kit (AP-MN-BL-GDNA-250, Axygen Biosciences, Union City, USA). Polymerase chain reactions (PCR) were carried out on genomic DNA (10 ng) using a TaqMan universal PCR master mix (Applied Biosystems). Forward and reverse primers and FAM and VIC labelled probes were designed by Applied Bio systems (ABI Assay-by-Designs) in a 5ul reaction. Sequences of primers and probes are available on request. Amplification conditions on BIO-RAD S1000^™^ thermal cyclers (Bio-Rad Laboratories, Hercules, California) were as follows: 1 cycle of 95°C for 10 min, followed by 40 cycles of 95°C for 15 s and 60°C for 1 min. The completed PCR products were read on an ABI PRISM 7900 Sequence Detector in end-point mode using the Allelic Discrimination Sequence Detector Software V2.3 (Applied Biosystems). For the software to recognize genotypes, two non-template controls were included in each 384-well plate. All patients’ and normal control samples were arrayed together in four 384-well plates and the fifth plate contained eight duplicate samples from each of four plates to ensure the quality of genotyping (the concordance was >99% for all SNPs).

### Statistics analysis

For each polymorphism, the deviation of genotype frequencies in controls from those expected under Hardy-Weinberg equilibrium was assessed by a goodness-of-fit x^2-^test. Linkage disequilibrium (LD) between pairs of biallelic loci was determined using two measures, D’ and r^2^. Either Chi-square test or Fisher's exact test was performed by comparing distributions of genotype frequencies between patients and controls. Risks associated with rare genotypes were estimated as odds ratios (ORs). Corresponding 95% confidence intervals (CIs) by an unconditional logistic regression were adjusted by age (scale variable) and sex (nominal variable). For haplotypes with frequencies >1%, risks were compared to the reference haplotype(major haplotype in control group)using an unconditional logistic regression model with the HAPSTAT 3.0 software, according to Lin et al [Copyright (c) 2006–2008 Tammy Bailey, Danyu Lin and the University of North Carolina, NC, USA.] [25,26]. HAPSTAT allows the user to estimate or test haplotype effects and haplotype-environment interactions by maximum likelihood estimation and the EM algorithm. All statistical tests were two-tailed and *P*-values were considered to be statistically significant when ≦0.05. All analyses were performed using a statistical analysis software for windows, version 9.2 (SAS Institute, Cary, NC, USA). The statistical power calculations were performed using the QUANTO software program (Version 1.2.3).

## Results

### Characteristics of subject allele frequencies of the htSNPs

A total of 414 patients gastric cancer (300 males and 114 females) aged 35 to 80 years old were included in this study. Of 1080 examinees, 148 individuals were screened for gastric atrophy using serum PG examination; 109 patients were confirmed with gastric atrophy by biopsy and histopathological examinations; 17 people were diagnosed as pseudopositive for gastric atrophy and excluded from the study; 22 people who rejected endoscopic examination were excluded from the study. Nine hundred and thirty-two individuals were included in the control group. Nine persons were excluded, due to the failure to detected anti-*H. pylori* Ig G and/or genotyps in blood samples. Finally, 923 subjects were included and analysed as control. The characteristics of subjects are summarized in Table 1. The mean age was older in gastric cancer patients than that in controls (61.5 vs. 50.7 years; *P* <0.001). There were more females in the control group (*P* <0.001). Prevalence of *H. pylori* seropositivity were significantly higher in groups of gastric cancer and gastric atrophy than that in the healthy control (70.3% vs. 49.7%, *P* <0.001; 75.2% vs. 49.7%, *P* = 0.01).

**Table 1:** **Characteristics of the subjects and the *PTPN11 *polymorphisms**

	**Gastric cancer (%)**	**Gastric atrophy (%)**	**Control(%)**	***P* value (%)**
N	414	109	923	
Sex				
Male	300(72.5)	64(58.7)	539(58.4)	<0.001
Female	114(27.5)	45(41.3)	384(41.6)	
Age				
≤45	32(7.7)	19(17.4)	276(29.9)	<0.001
46-65	224(54.1)	80(73.4)	579(62.7)	
>65	158(38.2)	10(9.2)	68(7.4)	
Anti-*H.pylori* IgG				
Negative	123(29.7)	27(24.8)	464(50.3)	<0.001
Postive	291(70.3)	82(75.2)	459(49.7)	
SNPs				
rs2301756				
GG	305(73.7)	86(78.9)	687(74.4)	0.462
GA	95(22.9)	22(20.2)	216(23.4)	
AA	14(3.4)	1(0.9)	20(2.2)	
rs12423190				
TT	214(51.7)	55(50.5)	476(51.6)	0.047
TC	166(40.1)	36(33.0)	373(40.4)	
CC	34(8.2)	18(16.5)	74(8.0)	
rs12229892				
GG	137(33.1)	40(36.7)	308(33.4)	0.963
GA	205(49.5)	50(45.9)	454(49.2)	
AA	72(17.4)	19(17.4)	161(17.4)	
rs7958372				
TT	305(73.7)	87(79.8)	685(74.2)	0.747
TC	102(24.6)	21(19.3)	222(24.1)	
CC	7(1.7)	1(0.9)	16(1.7)	
rs4767860				
AA	137(33.1)	39(35.8)	306(33.2)	0.386
AG	206(49.8)	44(40.4)	453(49.1)	
GG	71(17.1)	26(23.9)	164(17.8)	

**Figure 2:**
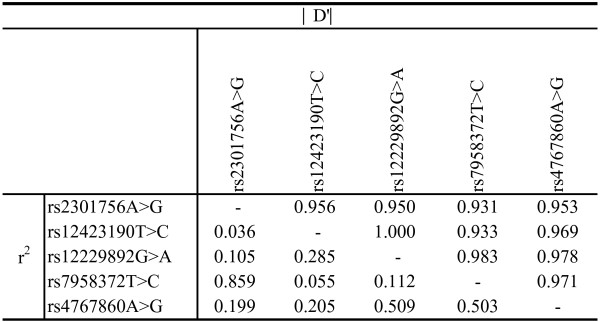
**Linkage disequilibrium coefficients (∣D’∣ and r2) between *PTPN11 ht *SNPs.**

The linkage disequilibrium structure of the 5 polymorphic loci is shown in Figure 2. All D’ values are above 0.93, and the pair-wise comparison among rs12423190 and rs12229892 revealed a complete LDs (∣D’∣ = 1 and r^2^ = 0.285). The genotype distribution for five htSNPs in the control group were in Hardy-Weinberg equilibrium (*P* value were 0.54, 0.94, 0.78, 0.68 and 0.87 respectively). In the present study, the distribution of rs12423190 genotype was found to be statistically different between groups (*P* = 0.047), however, distributions of rest four SNPs were not significantly different between groups.

### Association between htSNPs and *H. pylori* seropositivity, gastric atrophy and gastric cancer

The age and sex-adjusted ORs of gastric atrophy among *H. pylori* seropositive subjects were 0.93 (95%CI 0.55-1.57, *P* = 0.78) for CT and 2.20 (95%CI 1.06-4.55, *P* = 0.03) for CC genotype compared with rs12423190 TT genotype. In a recessive model, the age- and sex-adjusted OR was 2.47 (95%CI 1.13-4.55, *P* = 0.02) for CC genotype versus rs12423190 CT/TT genotypes (Table 2). However, the age and sex-adjusted ORs of gastric cancer among *H. pylori* seropositive subjects was 1.18 (95%CI 0.64-2.18, *P* = 0.60) for CT and 1.30 (95%CI 0.93-1.83, *P* = 0.13) for CC genotype compared with rs12423190 TT genotype. The haplotypes with frequencies ≥1% are shown in Table 2. The most frequent haplotypes were GTATA (rs2301756G, rs12423190T, rs12229892A, rs7958372T, rs4767860A) and four major haplotypes were accounted for over 95% of distribution. There was no significant association between haplotypes and gastric atrophy. The OR of GCGTG haplotype was 1.36 (95%CI 0.92-2.02, *P* = 0.12) versus the GTATA haplotype (Table 2). The association between the SNPs of *PTPN11* and the *H. pylori* infection was examined in health controls (Table 3), but no significant association between five htSNPs of *PTPN11* and *H. pylori* seropositivity was found. Distributions of *PTPN11* haplotypes were not correlated with *H. pylori* seropositivity. No association between htSNPs and gastric atrophy in *H. pylori* seronegative subjects was found either (data not shown). In a subgroup analysis, we did not find any association of htSNPs with Lauren’s classification, tissue differentiation and TNM staging (data not shown).

**Table 2:** ***PTPN11 *polymorphisms among *H. pylori *(+) subjects**

**Genotype**	**Control (%)**	**Gastric atrophy (%)**	OR(95%CI)^*^	***P*value**	**Gastric cancer (%)**	OR(95%CI)^*^	***P*value**
	**n = 459**	**n = 82**			**n = 291**		
rs2301756							
GG	74.3	76.8	Reference		73.9	Reference	
GA	23.5	22.0	0.90(0.51-1.59)	0.72	37.9	0.92(0.62-1.35)	0.66
A/A	2.2	1.2	0.51(0.06-4.09)	0.53	3.4	1.26(0.47-3.37)	0.65
rs12423190							
TT	52.5	50.0	Reference		50.9	Reference	
TC	40.1	34.1	0.93(0.55-1.57)	0.78	41.2	1.18(0.64-2.18)	0.60
CC	7.4	15.9	2.20(1.06-4.55)	0.03	7.9	1.30(0.93-1.83)	0.13
TT/TC	92.6	84.1	Reference		92.1	Reference	
CC	7.4	15.9	2.47(1.13-4.55)	0.02	7.9	1.21(0.89-1.76)	0.15
rs12229892							
GG	32.2	36.6	Reference		30.6	Reference	
GA	51.2	47.6	0.82(0.49-1.39)	0.46	52.2	1.04(0.72-1.51)	0.82
AA	16.6	15.9	0.86(0.42-1.75)	0.67	17.2	1.02(0.63-1.67)	0.93
rs7958372							
TT	74.1	78.0	Reference		74.2	Reference	
TC	24.4	20.7	0.80(0.45-1.43)	0.46	24.1	0.94(0.64-1.37)	0.74
CC	1.5	1.2	0.83(0.10-7.01)	0.86	1.7	1.16(0.30-4047)	0.83
rs4767860							
AA	33.8	34.1	Reference		31.9	Reference	
AG	49.5	40.2	1.54(0.82-2.91)	0.18	51.9	1.04(0.70-1.88)	0.59
GG	16.8	25.6	0.80(0.46-1.38)	0.43	16.2	1.32(0.92-1.90)	0.14
Haplotpe							
GTATA	41.3	39.6	Reference		41.2	Reference	
GCGTG	26.0	32.3	1.36(0.92-2.02)	0.12	27.6	1.12(0.80-1.57)	0.51
GTGTA	16.0	14.0	0.96(0.58-1.61)	0.89	14.4	1.14(0.88-1.47)	0.32
ATGCG	12.4	11.6	1.03(0.59-1.78)	0.92	13.1	0.96(0.71-1.32)	0.82
Others	4.3	2.5			3.7		

^*^OR for each genotype and hapolotype were calculated by age and sex adjusted logistic regression model.

**Table 3:** ***PTPN11 *polymorphisms for *H. pylori *seropositivity in control**

**Genotype**	**Hp + (%)**	**Hp-(%)**	OR(95%CI)^*^	***P*value**
	**(n = 459)**	**(n = 464)**		
rs2301756				
GG	74.7	75.2	Reference	
GA	23.3	22.8	1.18(0.51-2.72)	0.71
AA	2.0	2.0	1.52(0.49-4.66)	0.47
rs12423190				
T/T	52.1	50.7	Reference	
T/C	39.2	40.1	0.98(0.55-1.76)	0.95
C/C	8.7	9.2	0.92(0.29-2.93)	0.89
rs12229892				
G/G	32.9	34.6	Reference	
G/A	50.6	46.8	1.25(0.93-1.70)	0.15
A/A	16.5	18.5	1.01(0.65-1.57)	0.97
rs7958372				
T/T	74.7	74.9	Reference	
T/C	23.8	23.2	0.84(0.40-1.77)	0.65
C/C	1.5	1.8	0.61(0.13-2.79)	0.53
rs4767860				
A/A	33.8	33.0	Reference	
A/G	48.1	48.3	1.11(0.36-3.45)	0.86
G/G	18.1	18.7	1.00(0.55-1.84)	0.99
Haplotpe				
GTATA	41.0	41.6	Reference	
GCGTG	26.9	28.4	0.97(0.79-1.20)	0.77
GTGTA	15.7	14.8	1.08(0.83-1.40)	0.56
ATGCG	12.3	13.0	0.94(0.71-1.24)	0.66
Others	4.1	2.2		

^*^OR for each genotype and hapolotype were calculated by age and sex adjusted logistic regression model.

## Discussions

CagA-secreting *H*. *pylori* infection plays an important role in gastric carcinogenesis via a sequential CagA signal transduction pathway. CagA initially binds to seven protein components to activate aberrant cellular responses that promote the development of gastric cancer. Since the function of CagA protein is regulated by its binding partners, therefore genes that encode CagA interacting molecules may modify the risk of gastric cancer. In the present study, 5 htSNPs of the *PTPN11* gene were investigated for their associations with *H. pylori* infection, gastric atrophy and gastric cancer in Chinese Hans population. We found that subjects bearing rs12423190 CC genotype at intron 6 had a significantly higher risk in *H. pylori*-seropositive gastric atrophy. The OR for gastric cancer was increased in those carrying the GCGTG haplotype versus the GTATA (OR = 1.30, 95%CI: 0.93-1.83), however, the *P* value was 0.09, not statistically significant. In this study, the allele frequencies of rs12423190 were 71.8% for the T allele and 28.2% for the C allele among control subjects. The frequency of the C allele was similar to that reported in the HapMap Project (31.4%). Compared to the NCBI SNP database, we found that the frequency of the C allele is obviously higher in Chinese population than other populations (23.3% in Japanese; 11.5% in Utah residents with Northern and Western European ancestry). As incidences of gastric cancer are high in Chinese, Japanese and Koreans, high frequencies of this polymorphism in Asian populations may be part of explanation. Our findings indicated that the C allele contributes to genetic predisposition to *H. pylori*-induced gastric atrophy in Chinese population. A further study is required to confirm associations of the *PTPN11* rs12423190 polymorphism with gastric atrophy in diverse ethnic populations.

Recent studies revealed significant associations between the rs2301756 AA genotype in intron3 and reduced risk of gastric atrophy among *H. pylori*-seropositive Japanese subjects [7,19,22]. Our study found a similar trend of decreased risk of OR for rs2301756, however, the OR of atrophy was not significantly lower compared with the GA genotype (OR = 0.90, 95%CI: 0.51-1.59) and AA genotype (OR = 0.51, 95%CI: 0.06-4.09). In controversy, the association study for rs2301756 and gastric atrophy showed a completely opposite result in Uzbekistan population [20]. Zhu F and colleagues also demonstrated that the rs2301756 A allele was associated with low risk of intestinal metastasis (IM) indicating that *H. pylori* infection induces gastric precancerous lesions, such as gastric atrophy (OR = 0.46, 95%CI: 0.21-0.99) in *H. pylori* seropositive individuals. Meantime, an inverse association was shown in *H. pylori*-seronegative subjects in the same study (OR = 2.51, 95%CI: 1.21-4.43) [27]. There were no statistically differences in the frequency of rs2301756 in our study and others [21,28]. Inconsistent results from different studies may be due to different environmental backgrounds and ethnic groups. In our study, gastric atrophy was only identified in 82 cases of *H. pylori*- seropositive subjects, the minor allele of rs2301756 was relatively rare (MAF = 0.139). The statistical power maybe not sufficient (power = 0.10) to examine the association. A further investigation of association between rs2301756 and gastric atrophy is needed in a large population and other ethnic groups.

The functions of *PTPN11* polymorphisms are still unknown. SHP-2 contains two tandem SH2 domains, a PTP domain, and other functional motifs. Genetic mutations in PTPN11 exon 3(encoding the N-SH2 domain), exon 4(encoding the C-SH2 domain), Exons 7, 8 and 12(encoding the PTP domain) have been identified in Noonan syndrome, juvenile myelomonocytic leukemia, LEOPARD syndrome, lung cancer, liver cancer and colon cancer [29]. The interaction of tyrosine phosphorylated Cag-A with the SH2 domain of the protein is supposed to induce a conformational change in SHP-2 that weakens the inhibitory interaction between PTP and N-SH2 domain, and results in activation of SHP-2's catalytic activity [10,30]. The rs12423190 polymorphism is located in the intron 6, 1408 bp upstream exon 7, encoding part of PTP domain. Using a free bioinformatic tool (http://fastsnp.ibms.sinica.edu.tw/pages/inputCandidateGeneSearch.jsp) the rs12423190 is predicted to locate in the side of an intronic enhancer which could affect the gene transcriptional regulation. SHP-2 has several biological functions. The gain-of-function of SHP-2 may accelerate the downregulation of T-cell and B-cell activation through CTLA-4/PD-1 as well as IL-6/STAT3 signalings, eventually leading to a decrease in inflammation. Meantime, it might act as a signal promoter in inflammation. SHP-2 promotes growth factor induced activation of phosphatidylinositol 3-kinase (PI3-K)/Akt, the extracellular signal-related kinases (ERKs) and nuclear factor-kappa B (NF-κB). SHP-2 can either negatively or positively regulate the activation of Janus kinase 2 (Jak2)/signal transducer and activator of transcription (STAT) and the c-Jun-amino terminal kinases (JNKs) depending on different circumstances. rs12423190 may promote the over-expression of SHP-2, further involvement in the up-regulation of inflammatory cytokines through MAPKs and NF-κB signaling pathways, eventually leading to increase in inflammation related to atrophy [31]. However, the rs2301756 does not appear to reside in transcription factor binding sites or splicing sites, but is in the LD with associated haplotypes. The rs12423190 polymorphism is also in a linkage disequilibrium, which may contain other unidentified causative SNPs. Further studies of *PTPN11* sequence variants and their biologic functions may shed light in understanding the association of *PTPN11* polymorphisms and the risk of GA.

In addition, we demonstrated that gastric cancer patients had high prevalence of both *H*. *pylori* seropositivity and gastric atrophy; the prevalence of *H*. *pylori* seropositivity was significantly higher in subjects with gastric atrophy compared to controls without gastric atrophy, implying the important links with of *H*. *pylori* infection, gastric atrophy, and gastric cancer. Genetic factors, such as *PTPN11* polymorphisms may contribute to gastric atrophy, through affecting the connection of Cag A and SHP-2 protein.

The first limitation in our study is the *H*. *pylori* infection status being determined by serology, not by the serum CagA antibody test. In East Asia countries, almost all *H. pylori* strains reported from infected patients were East Asian CagA positive strains [32-34], therefore *H. pylori* strains in infected patients most likely possess CagA in our study. The second limitation is the PG criteria for GA screening, the criterion for GA is PGI <82.3 ng/ml and PGI/II ratio <6.05. These parameters for atrophy are used in China and have been validated in only one histological confirmatory study [23], it is may be insufficient to draw a reliable diagnosis, thus more confirmatory studies are needed. Finally, a small sample size of gastric atrophy, especially for the cohort of *H*. *pylori* (+) gastric atrophy individuals is due to a low incidence of gastric atrophy in China. A large-scale study for recruiting more patients with gastric atrophy is required to confirm our findings in the future.

## Conclusions

In conclusion, our study provides the first evidence that rs12423190 polymorphism of the *PTPN11* gene is significantly associated with an increased risk of gastric atrophy in *H. pylori* infected Chinese population, suggesting that rs12423190 polymorphism could be used as a biomarker of genetic susceptibility to gastric atrophy.

## Competing interests

No competing interests to be disclosed.

## Authors’ contributions

JJ and CX designed and carried out most of study; JZF, KF, JMS_,_ WYP and TSY participated data acquisition and analysis; JJ and JZF wrote the first draft of manuscript. All authors contributed to and approved the final manuscript by providing constructive suggestions.

## Pre-publication history

The pre-publication history for this paper can be accessed here:

http://www.biomedcentral.com/1471-230X/12/89/prepub
